# Multiphysics Simulation of Low-Amplitude Acoustic Wave Detection by Piezoelectric Wafer Active Sensors Validated by In-Situ AE-Fatigue Experiment

**DOI:** 10.3390/ma10080962

**Published:** 2017-08-17

**Authors:** Md Yeasin Bhuiyan, Victor Giurgiutiu

**Affiliations:** Department of Mechanical Engineering, University of South Carolina, Columbia, SC 29208, USA; victorg@sc.edu

**Keywords:** structural health monitoring, acoustic wave, AE signal, sensor, multiphysics simulation

## Abstract

Piezoelectric wafer active sensors (PWAS) are commonly used for detecting Lamb waves for structural health monitoring application. However, in most applications of active sensing, the signals are of high-amplitude and easy to detect. In this article, we have shown a new avenue of using the PWAS transducer for detecting the low-amplitude fatigue-crack related acoustic emission (AE) signals. Multiphysics finite element (FE) simulations were performed with two PWAS transducers bonded to the structure. Various configurations of the sensors were studied by using the simulations. One PWAS was placed near to the fatigue-crack and the other one was placed at a certain distance from the crack. The simulated AE event was generated at the crack tip. The simulation results showed that both PWAS transducers were capable of sensing the AE signals. To validate the multiphysics simulation results, an in-situ AE-fatigue experiment was performed. Two PWAS transducers were bonded to the thin aerospace test coupon. The fatigue crack was generated in the test coupon which had produced low-amplitude acoustic waves. The low-amplitude fatigue-crack related AE signals were successfully captured by the PWAS transducers. The distance effect on the captured AE signals was also studied. It has been shown that some high-frequency contents of the AE signal have developed as they travel away from the crack.

## 1. Introduction

### 1.1. State of the Art

The structural health monitoring (SHM) technology is increasingly used for the detection of progressive defects in the critical aerospace structures [1,2,3,4]. SHM has been introduced for both active and passive damage identification for the real-time monitoring of the structures. Monitoring of the acoustic emission (AE) from the progressive fatigue damage is categorized as the passive online monitoring [5,6,7]. The AE signals from the fatigue crack have always been an interest for the researchers [8,9,10,11]. Lee et al. experimentally showed that the AE waves from fatigue crack growth propagate as guided waves [8]. They used the commercially available resonant type (250 kHz) and wideband (100 kHz–600 kHz) AE sensors to capture the AE signals. These sensors were designed to effectively measure the out-of-plane wave motion. However, the AE signals from the fatigue crack are usually low-amplitude signals and therefore challenging to detect using the conventional AE sensors [12,13].

Piezoelectric AE sensors such as PICO, S9225, WSA had been used to detect the high-amplitude simulated AE signals [14,15]. They were also used for detecting the AE signals from the pencil lead break (PLB), impact damage [16,17]. However, the AE signals from these sources were generally high-amplitude signals. They had higher-amplitude out-of-plane components and these AE sensors were also well-constructed to sense the out-of-plane wave motion. Thus, the AE sensors could effectively sense the high-amplitude AE signals from these sources. However, the AE signals due to the fatigue crack in the thin plate-like aerospace structures are much more complex than the simple PLB and impact damage [18,19,20,21]. 

AE hits from the crack in a notched thick specimen were captured by the piezoelectric AE sensors [12]. However, the surface waved were dominant in these AE signals. The statistical Bayesian approach was developed to analyze the AE hits by Agletdinov et al. [22]. Nam and Mal [23] identified three types of elastic during fatigue crack in the aluminum specimen. However, no load information was mentioned about at what load level the AE signals had happened. Andreykiv et al. analytically showed that the formation of a penny-shaped crack in the aluminum alloys emits acoustic waves [24]. They simulated different modes of crack formation by a sudden drop of the stress on the crack surface. 

The experimental analyses supported by numerical simulations provide deeper insight of the elastic wave propagation phenomena [25,26,27]. Willberg et al. [28] reviewed the research on guided wave simulations and compared various numerical methods. They provided a comprehensive list of references for the simulation of guided waves. They showed that depending on the application certain methods could be more appropriate than others. The higher order finite element method (FEM) for Lamb wave had been performed [29]. Gresil et al. [30] investigated the general guidelines for Lamb wave modeling as referred in the review paper as well. These research provided very important guidelines that have been followed in the present research. However, the fatigue crack related AE source modeling had not been explicitly investigated in these studies. 

Many theoretical works were developed to correlate the AE waveforms to their sources [31,32,33]. In seismology, the moment tensor approach was used to describe the movement of a fault during an earthquake [34,35,36]. The moment tensor comprises with nine generalized couples including three dipoles. Buried monopole and dipole with various step functions and fracture mechanics-based methods were used to model the AE source [37,38,39]. However, the interaction of the AE waveform to the crack boundary in a thin plate is yet to be analyzed. The finite element (FE) methods are the most effective tool to estimate the acoustic wave field [28,40].

Piezoelectric wafer active sensors (PWAS) were commonly used for SHM applications [41,42]. These sensors successfully captured the guided waves in active detection technique [43,44]. The active detection method required an user-defined excitation signal where the high-amplitude signals can be used to excite the structure.

### 1.2. Principles of the PWAS Transducer

**Figure 1:**
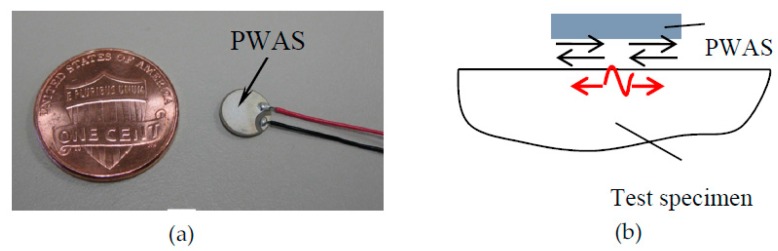
(**a**) Piezoelectric wafer active sensor (PWAS) [45]; (**b**) acoustic waves sensing mode of the PWAS (in-plane and out-of-plane wave motion sensing capability of the PWAS).

PWAS transducers are small, lightweight, inexpensive, and can be produced in different geometries [45]. They can be permanently bonded on host structures in large quantities and achieve real-time monitoring of the structural health status. They can be conveniently used for transmitting and receiving guided waves. A PWAS mounted on the structure is shown in Figure 1a. The sensing modes of a PWAS are illustrated in Figure 1b. It can measure both symmetric (wave motions are predominantly in-plane) and antisymmetric Lamb wave modes (wave motions are predominantly out-of-plane).

PWAS transducer couples the electrical and mechanical effects (mechanical strain, $S_{ij}$, mechanical stress, $T_{kl}$, electrical field, $E_{k}$, and electrical displacement, $D_{j}$). The piezoelectric constitutive equations in tensor notations can be written as

(1) $$S_{ij}=s_{ijkl}^{E}T_{kl}+d_{kij}E_{k}$$

(2) $$D_{j}=d_{klj}T_{kl}+\varepsilon_{jk}^{T}E_{k}$$

where $s_{ijkl}^{E}$ is the mechanical compliance of the material measured at zero electric field (E=0), $\varepsilon_{jk}^{T}$ is the dielectric permittivity measured at zero mechanical stress (T=0), and $d_{klj}$ represents the piezoelectric coupling effect. PWAS utilizes coupling between in-plane strains $S_{1},S_{2}$ and transverse electric field $E_{3}$. The $d_{31}$ coupling is the main effect but other couplings maybe present due to manufacturing limitations. However, the influences of the other couplings on the voltage signals are relatively smaller.

The AE signals have much lower amplitude than the typical guided waves generated by the PWAS transducer for active sensing applications. The suit of the PWAS transducers in application to low-amplitude fatigue-crack related AE signals has yet to be done. Assessment of the PWAS transducer to capture the AE signals using multiphysics FEM simulation and experiment would be an interest in the SHM and NDE community.

### 1.3. Scope of the Article

The novelty of the article consists in using PWAS transducers to capture AE signals generated by an advancing crack during cyclic fatigue loading. In the past, PWAS transducers had been used for active SHM sensing where a PWAS transmitter is excited with a relatively large electric tone burst (10–20 V amplitude) and the resulting elastic waves are captured by a PWAS receiver. In the present work, we have used only PWAS receivers during passive SHM sensing to capture the elastic waves generated by AE events during fatigue cyclic loading of a cracked specimen. The elastic waves generated by AE events are of relatively much lower amplitude than those generated during active SHM sensing. We found that the AE signals are much weaker than the active SHM signals; the AE signals were also contaminated with environmental and electromagnetic noise. We had to implement denoising techniques to clean the AE signals and reveal their frequency content related to fatigue cracking as contained in the AE waveform [46]. A combination of multiphysics simulations and experiments were performed. Some important aspects of the AE signals have been obtained that could potentially be used for the fatigue crack length estimation. To validate the simulation results, in-situ AE-fatigue experiments were performed. The experimental results supported the simulation results. 

## 2. Description of the 3D Multiphysics FEM Simulation

The multiphysics simulations were performed on the three-dimensional finite element (FE) model by combining the physics of piezoelectric and structural materials. The wafer type piezoelectric sensors also known as PWAS were simultaneously modeled with the host structure to accomplish the multiphysics simulation. The transient FE analysis was performed to obtain the time-domain signals and the acoustic wave propagation in the structure. Aircraft grade aluminum 2024-T3 material properties were used for the host structure. The material properties of the plate were: modulus of elasticity of the material, E = 73.1 GPa; Poisson’s ratio, ν = 0.33, the density, ρ = 2780 kg/m^3^; and the shear wave speed, *c*_s_ = 3140 m/s. 

### 2.1. Practical Aspects of the Model

**Figure 2:**
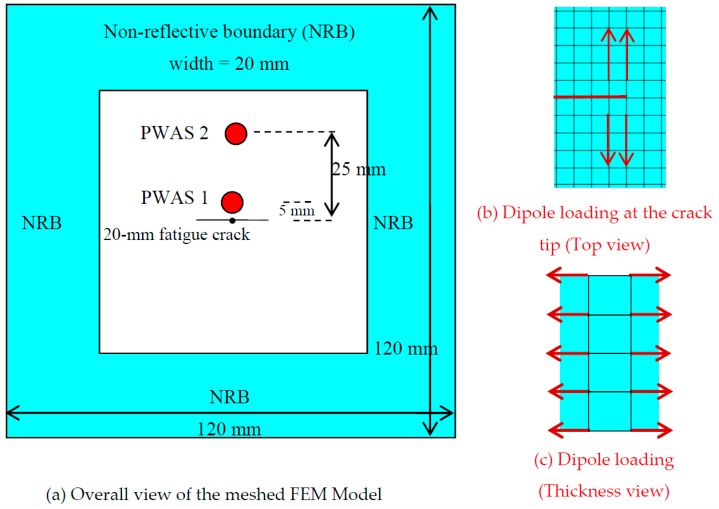
The meshed FEM model with in-plane dipole excitation: (**a**) overall view; (**b**) top view zoomed into crack tip area showing dipole loading; (**c**) Side view showing thickness wise assignment of dipole components.

The geometry of the model is shown in Figure 2. A thin (thickness = 1 mm) square plate with 120 mm side was modeled using ANSYS 15.0 (ANSYS, Inc., Canonsburg, PA, USA). Since it has been an interest for the fatigue crack related AE signal simulation, a fatigue crack was introduced in the plate. In practice, the fatigue crack always initiates from some kind of discontinuities such as holes, notch, joints etc. By keeping this in mind, a small 1-mm diameter hole was modeled in the center of the crack. The hole was modeled with a 9.5-mm fatigue crack on each side. The total length (2a) of the crack was 20-mm (tip to tip). The host structure was meshed with eight nodes SOLID45 structural element. This geometry was chosen by keeping in mind that we could run an AE-fatigue experiment to validate the simulation results.

### 2.2. Modeling of the Fatigue Crack

There were numerous ways to model an actual fatigue crack in FEM [47,48]. We adopted a popular and simpler method to model the crack. The crack was modeled using the discontinuity at the adjacent pair of nodes along the two crack surfaces. There were two sets of nodes along the crack faces and each set was representing the nodes on each face. The nodes are discontinuous along the crack faces and there was no interaction between these nodes. Two sets of nodes were adjacent to each other and the solid elements were disbanded along the crack faces. This type of modeling can represent a crack opening situation while crack grows near the peak load of the fatigue loading in practice. The modeling of the cracks using the above approach is fair enough to model the actual cracks with a small hole in the plate-like structure in the finite element methods [47,49]. This crack modeling approach had also been implemented in active SHM sensing [50]. The symmetric crack was modeled about the center small hole as shown in Figure 2a.

### 2.3. Modeling of PWAS Transducers

Two PWAS transducers were modeled together with the host aluminum structure. The size of each PWAS was 7-mm. One PWAS was modeled in the near-field of crack and the center of the PWAS was at 5-mm from the crack. The other PWAS was modeled in the far-field of crack and the center of the PWAS was at 25-mm from the crack. The PWAS was meshed with eight node SOLID5 coupled field element. This element with properly selected coupled field options can handle the physics of piezoelectric and structural materials. The properties of the PWAS were chosen based on the experimentally used PWAS transducers supplied from the manufacturer. The in-plane polarization was chosen for the modeled PWAS so that it could sense the in-plane strain of the host structure. The following material properties for the PWAS transducer had been used [51].

(3) $$C=\left[\begin{array}{cccccc} 97 & 49 & 49 & 0 & 0 & 0\\ 49 & 49 & 49 & 0 & 0 & 0\\ 49 & 49 & 84 & 0 & 0 & 0\\ 0 & 0 & 0 & 24 & 0 & 0\\ 0 & 0 & 0 & 0 & 22 & 0\\ 0 & 0 & 0 & 0 & 0 & 22 \end{array}\right]\cdot \mathrm{GPa}$$

(4) $$\varepsilon =\left[\begin{array}{ccc} 947 & 0 & 0\\ 0 & 605 & 0\\ 0 & 0 & 947 \end{array}\right]\times \;10^{-8}\cdot F/m$$

(5) $$e=\left[\begin{array}{cccccc} 0 & 0 & 0 & 0 & 12.84 & 0\\ 0 & 0 & 0 & 12.84 & 0 & 0\\ -8.02 & -8.02 & 18.31 & 0 & 0 & 0 \end{array}\right]\cdot C/m^{2}$$

where C is the elastic stiffness matrix, ε is the dielectric matrix, and e is the piezoelectric stress matrix.

The piezoelectric transduction used through the PWAS material properties directly gave the voltage signal as an output. Any mismatch due to meshing should be avoided for better FEM results. Thus the PWAS and the underneath host structure was meshed using the same topology of meshing. This ensured that each node of the PWAS element coincided with the corresponding node of the host structure element. The nodes between the PWAS elements and the structural elements are then merged together to simulate a perfect bonding between PWAS and host structure. However, in practice, there is a very thin adhesive layer in between the PWAS and host structure. The thickness of the adhesive layer depends on the operator and this variability is not considered in the present work. The effect of adhesive layer had been investigated by Santoni-Bottai [52], Lin et al. [53] in our LAMSS group. It is recommended to use a very thin layer of adhesive of better agreement between simulation and experimental signal.

### 2.4. Modeling of the Acoustic Emission Event at the Crack Tip

The acoustic emission source at the crack tip was modeled using the dipole concept as suggested by Hamstad et al. [37] and Prosser et al. [26]. The dipoles basically represent the components of the moment tensor as discussed by Aki and Richards [34]. The moment tensor components were used to simulate the seismological acoustic events. Two dipoles were used to simulate the AE event due to fatigue crack growth. The dipoles were applied at the tip of the crack as shown in Figure 2b. The dipoles were placed at one element apart from each other that represented a unit growth of the fatigue-crack. Since the crack in the thin plate usually happens along the entire thickness, the dipoles were placed along the thickness as line dipole source as shown in Figure 2c. 

**Figure 3:**
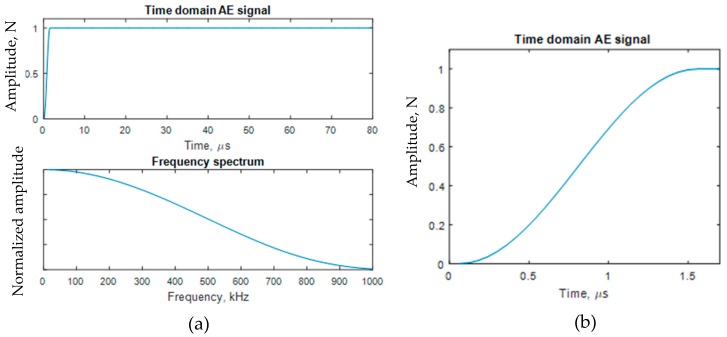
The dipole loading used for simulating the acoustic emission (AE) event at the crack tip. (**a**) A typical half-cosine bell step function and its frequency response (Rise time = 1.5 µs); (**b**) The half-cosine bell step-up from 0 to 1 in the amplitude scale.

Each dipole consisted of two self-equilibrating point forces of equal strengths. The magnitude of the point force follows a sharp rising step function. This type of step function represented a wideband AE source at the crack tip. The rise time was the characteristic parameter of the step function. The half-cosine bell rising step function with 1.5 μs rise time was used in the modeling following the Ref. [37]. The time variation and the frequency spectrum of the half-cosine bell step function are shown in Figure 3. This type of the smoothing step up function was used for the modeling since it provided a better simulation of the AE event as mentioned in Ref. [37]. The transient integration parameters were: γ = 0.0, α = 0.25, δ = 0.5, θ = 0.5, oscillation limit criterion = 0.5, time step = 0.12 µs. These values were carefully chosen to obtain numerical stability.

The element size of the model was selected such that the simulation result could predict a wide range of frequencies (40 kHz to 700 kHz). In general, 10–15 nodes per wavelength were considered throughout the model. The element sizes were varied in the model. The smaller elements were used in the high-stress gradient region especially near the crack, interface of the sensors and structure. The relatively larger element size was used outside the crack region. This resulted in more than one million (1.092 M) elements in the 3D space. The multiple FEM simulations were performed (not shown here) to obtain the convergence of the results. Four elements were used in the thickness direction. The meshing was used following the criteria mentioned in Ref. [37] to achieve better FEM results. The non-reflective boundary (NRB) was used in the FEM model to avoid the reflections from the plate edges. The NRB was modeled using the criteria mentioned in Ref. [54]. 

The aspect ratio of the element was maintained close to one which allowed a better accuracy in the results. The full integration method was used in the solution to avoid the hourglass effect as recommended by ANSYS. Also, with the proper refinement of meshing and maintaining reasonable aspect ratio, the shear locking effect has been diminished in the FEM results, but, may still be present in smaller amounts, since the full integration method was used. To completely avoid these artefacts, higher order elements or different finite element schemes as presented in Ref. [29] are suggested for further investigation.

## 3. Multiphysics FEM Simulation Results

The multiphysics simulation results showed that the acoustic event at the crack tip generated acoustic waves in the structures. These acoustic waves were captured by the both PWAS transducers as AE signals. The simulated results can be discussed in two significant aspects as follows:

### 3.1. Acoustic Emission Propagation and Secondary Source of AE Signals

**Figure 4:**
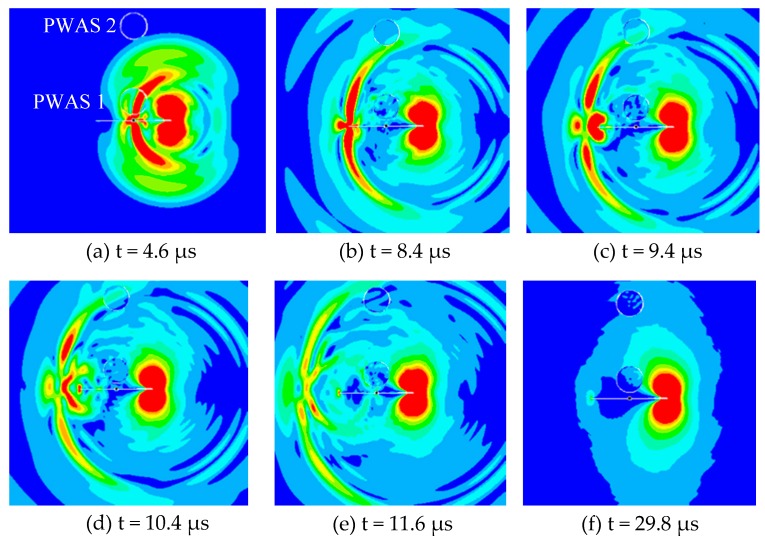
The animation snapshots of the acoustic wave propagation at different time of simulation: (**a**) the acoustic wave traveling toward the PWAS 1; (**b**) the waves reached to the PWAS 2 and the other tip of the crack; (**c**) the other crack-tip generated secondary AE event; (**d**) the reflected AE waves traveling back toward the first crack tip; (**e**) the reflected AE waves hit the PWAS 1 again; (**f**) after back and forth AE traveling along the crack and another secondary AE event at the other crack-tip.

The effect of the AE event at the crack tip propagated as the guided acoustic waves in the plate. The animation snapshots at various time of the simulation are shown in Figure 4. An AE event occurred at the one crack-tip, referred to as “primary AE event” and the generated AE waves travel in the plate. The acoustic waves hit the near-field PWAS 1 and then passed it as the time had progressed. Subsequently, the guided waves hit the far-field PWAS 2 and the other crack-tip. When the waves hit the other crack-tip it generated another AE event referred to as “secondary AE event”.

Both primary and secondary AE event may generate the AE signals that were captured by the two PWAS transducers. The local dynamics of the crack surface and the acoustic waves may generate a crack resonance as we verified by the experimental measurement in our recently published paper [55]. This local crack resonance phenomenon is highly related to the fatigue crack length. Thus it could potentially be used for fatigue crack length estimation.

It was observed that the acoustic waves propagate with Rayleigh surface wavespeed along the crack surface. The near-field PWAS may sense complex guided wave signals as it was placed near to the source. The far-field PWAS may sense the plate guided waves and some feature of the secondary AE event.

### 3.2. Simulated AE Signals Captured by Two PWAS Transducers

**Figure 5:**
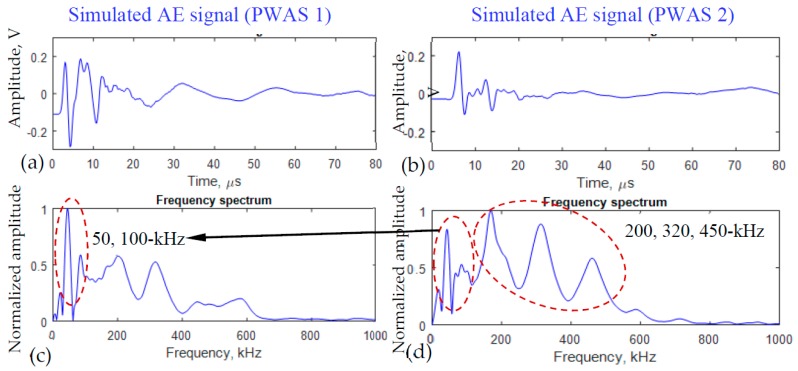
Multiphysics simulated AE signals from the two PWAS transducers. The typical AE signals coming from the same AE event captured by the (**a**) near-field PWAS 1; (**b**) far-field PWAS 2; (**c**,**d**) Some high-frequency contents (200, 320, 450-kHz) have relatively higher amplitude than the low-frequency component as the acoustic wave traveled away from the crack.

From the simulation, it was shown that both PWAS transducers captured the AE signals. The time domain signals of the two PWAS transducers are shown in Figure 5a,b. The fast Fourier transform (FFT) was performed to show the frequency spectra of the two signals. Figure 5c shows that the highest peak of the PWAS 1 signal is at 50-kHz frequency. The next consecutive peaks are located at 100, 200, 320-kHz. Figure 5d shows that the highest peak of the PWAS 2 signal is at 200-kHz frequency. The next consecutive peaks are located at 50, 100, 320, 450-kHz. The higher frequency contents such as 100, 200, 320, 450-kHz peaks gained amplitude as compared to the 50-kHz frequency peak. It may be because these high-frequency signals may become fully-developed as they travel away from the source.

### 3.3. Simulated AE Signals with Two PWAS Transducers on the Two Sides of the Crack

**Figure 6:**
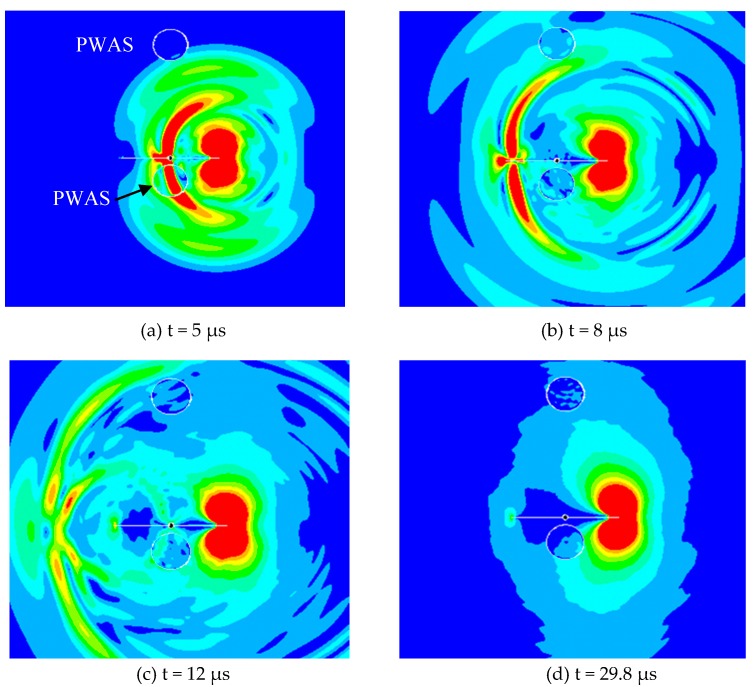
The animation snapshots of the acoustic emission signal propagation when the two PWAS transducers were on the two sides of the crack. The near PWAS 1 was at 5-mm from the crack and the far PWAS 2 was at 25-mm from the crack but on the other side of the crack. The traveling fatigue-crack-generated AE signals at different time of simulation are illustrated.

A new FEM model has been set up to study any dynamic characteristics, mode conversion or shadowing effect on the far PWAS due to the obstruction of the near PWAS. In this case, the far PWAS was kept same as the previous case but the near PWAS was modeled on the other side of the crack. The relative position of the two PWAS transducers with respect to the fatigue crack is illustrated in Figure 6. In this new configuration of the sensors, it was expected that the far-field PWAS signal would not be affected by the near-field PWAS. 

The animation snapshot of the acoustic wave propagation at different time of the simulation is illustrated in Figure 6. The wave propagation phenomenon is almost same as the previous situation (Figure 4). The three types of guided waves were observed: crack surface waves, the plate guided Lamb waves and SH waves. The crack resonance feature was also observed in this case. The simulated AE signals were recorded by the two PWAS transducers.

### 3.4. Comparison between the Two Simulated AE Signals Corresponding to the Two Sensor Configurations

**Figure 7:**
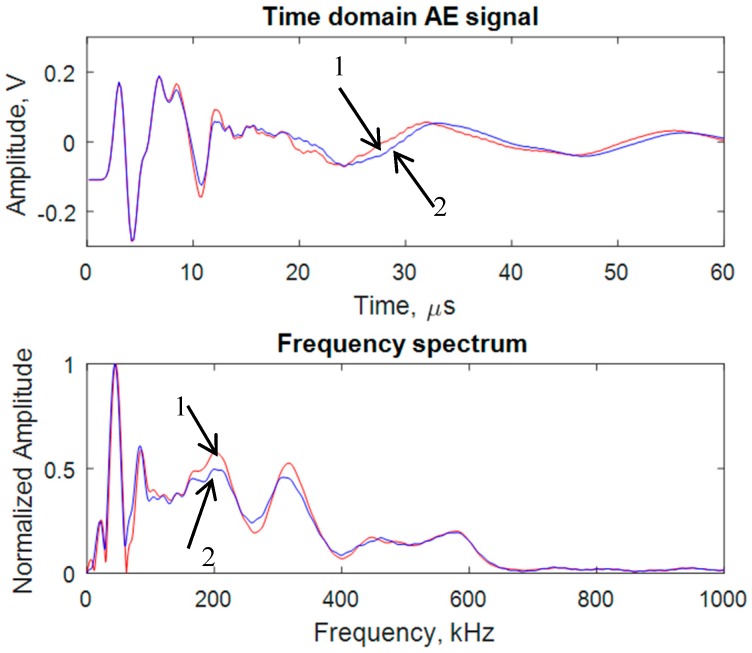
Simulated AE signals captured by the near PWAS 1 for two different sensor configurations: 1 → both sensors are on the same side of the crack, 2 → Sensors are on the opposite side of the crack.

Two sensor configurations were considered. Configuration 1: the two sensors were on the same side of the crack as illustrated in Figure 2a; configuration 2: the two sensors were placed on two sides of the crack (Figure 6). These configurations are referred to as “1” and “2”. The AE waveforms and their frequency spectra of the near-field PWAS signals are shown in Figure 7. It indicates that the both signals are almost same as expected. Hence, the simulated AE signals were consistent with each other.

**Figure 8:**
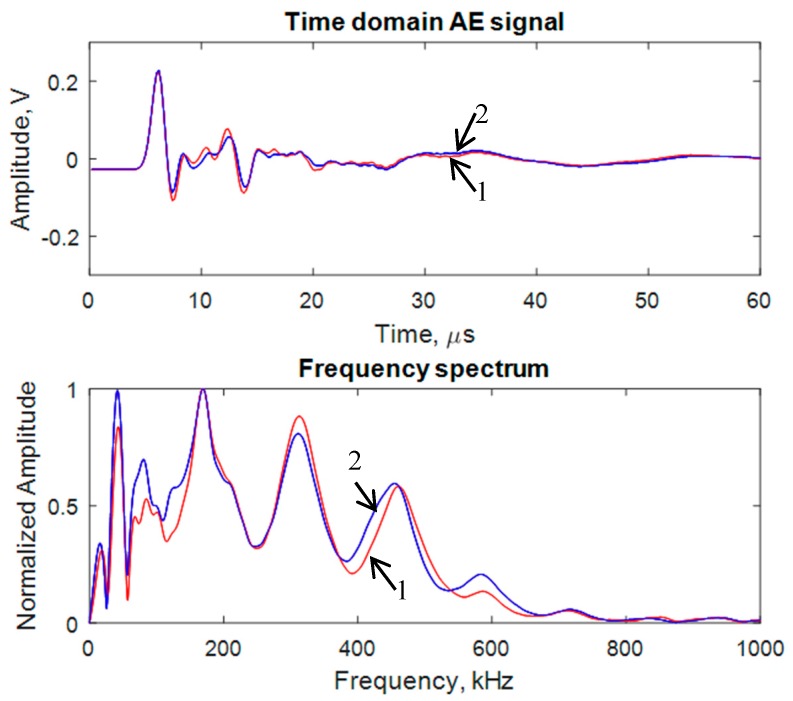
Simulated AE signals captured by the near PWAS 1 for two different sensor configurations: 1 → both sensors are on the same side of the crack, 2 → Sensors are on the opposite side of the crack.

The AE waveforms and their frequency spectra of the far-field PWAS signals are shown in Figure 8. The waveforms and frequency spectra were very similar to each other. Hence, we could conclude that the near-field PWAS had very minimal effect on the dynamic characteristic of the far-field PWAS.

### 3.5. Simulated AE Signals with Individual PWAS Transducer

We also conducted FEM simulations by placing one sensor at a time (not shown here). The similar results as discussed in Section 3.3 and Section 3.4 were obtained. As far as the two sensors were sufficiently far from each other, the one sensor dynamics has no effect on the dynamics of another sensor.

## 4. Description of the In-Situ AE-Fatigue Experimental Setup

**Figure 9:**
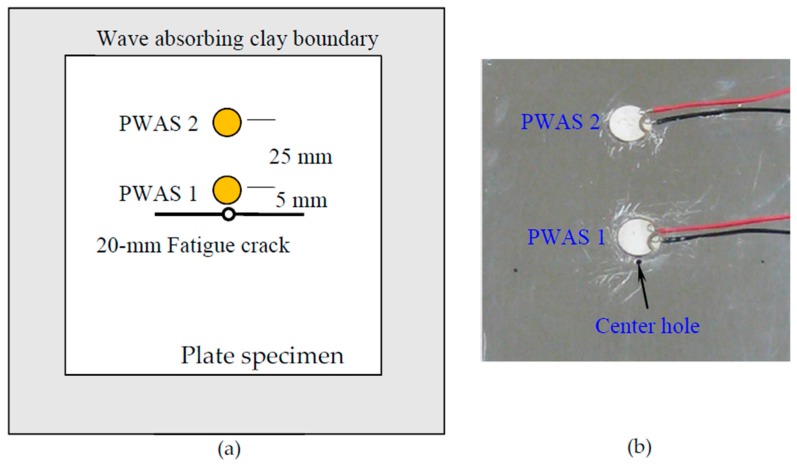
(**a**) Schematic diagram of the specimen with fatigue crack and a PWAS transducer bonded at 5-mm and 25-mm from the crack; (**b**) Actual specimen with two PWAS transducers bonded at 5-mm and 25-mm from the crack.

To validate the multiphysics simulation results, an in-situ AE-fatigue experiment was designed using two PWAS transducers. The AE signals emanated from the fatigue crack was measured with simultaneous measurement of the fatigue loading. Aircraft grade aluminum Al-2024 T3 material was used to make the specimen. The dimension of the specimen was 305 mm in length, 100 mm in width, and 1 mm in thickness. Like the non-reflective boundary in the simulation, the wave-absorbing clay boundary was used in the experiment. The schematic diagram of the experimental setup is shown Figure 9a. Two PWAS transducers were bonded at 5-mm and 25-mm from the crack (Figure 9b) by mimicking the simulation configuration.

**Figure 10:**
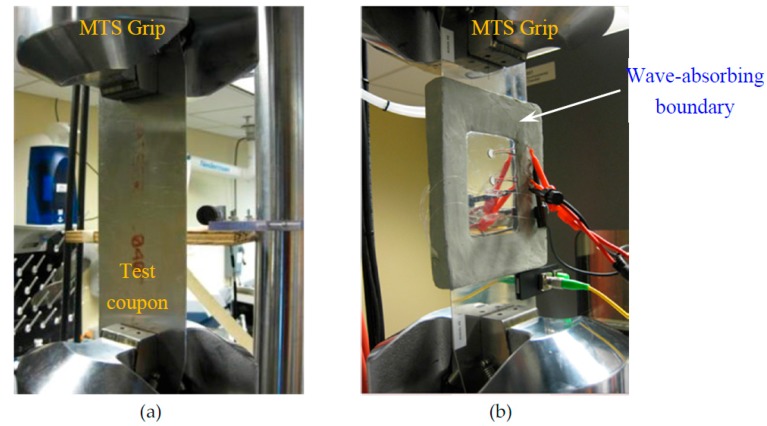
(**a**) Actual test coupon in the MTS grip without any AE instrumentation for the initial 20-mm crack; (**b**) Instrumented Test coupon in the MTS grip for capturing fatigue-crack related AE signals. Two PWAS transducers were used to capture the AE signals.

For the fatigue crack initiation, a small 1-mm hole is drilled at the center of the specimen and the cyclic fatigue loading was applied by using the MTS machine (MTS Systems Corporation, Eden Prairie, MN, USA). An axial tensile cyclic fatigue loading was varied sinusoidally between 2.3 kN to 23 kN. These load levels gave a stress level of 6.5% and 65% of the yield limit (345 MPa) of the material which is commonly used for practical aircraft testing for structural integrity [56]. A 20-mm fatigue crack was created to mimic the simulation condition. During the initial crack experiment, no AE instrumentation was employed as shown in Figure 10a. 

The specimen with 20-mm fatigue crack was then equipped with two PWAS transducers and wave absorbing clay boundary. The diameter of each PWAS transducer was 7 mm. The instrumented specimen was then subjected to fatigue loading as shown in Figure 10b. The use of wave absorbing boundary would provide cleaner AE signals without having any plate edge reflections.

Three measurement systems were used simultaneously during the latter stage of the experiment. They were: (a) AE signal measurement by the AE system; (b) MTS load application by the MTS system; and (c) fatigue crack growth measurement by the high-resolution video recording. The load level was reduced to the 60% of the previous load level and the cyclic loading was applied slowly (0.05 Hz). This loading frequency had no interference with the captured AE signals. Also, a band-pass filter (30 kHz–700 kHz) was used to avoid any interference from the low-frequency noises such as hydraulic loading, MTS grips, and mechanical vibrations. A 40 dB preamplifier was also used with the band-pass filter which was recommended by the manufacturer of the AE system. Under the axial cyclic loading, the fatigue crack grew from 20-mm to 25-mm. For the triggering of the AE signal measurement, a threshold had been chosen 2 dB above the environmental noise level.

## 5. AE-Fatigue Experimental Results

From the experimental results, we observed that the PWAS transducers successfully captured the AE signals coming from the fatigue crack. The experimental results can be discussed in two aspects as follows:

### 5.1. Fatigue Crack Related AE Hit Captured by the Two PWAS Transducers

**Figure 11:**
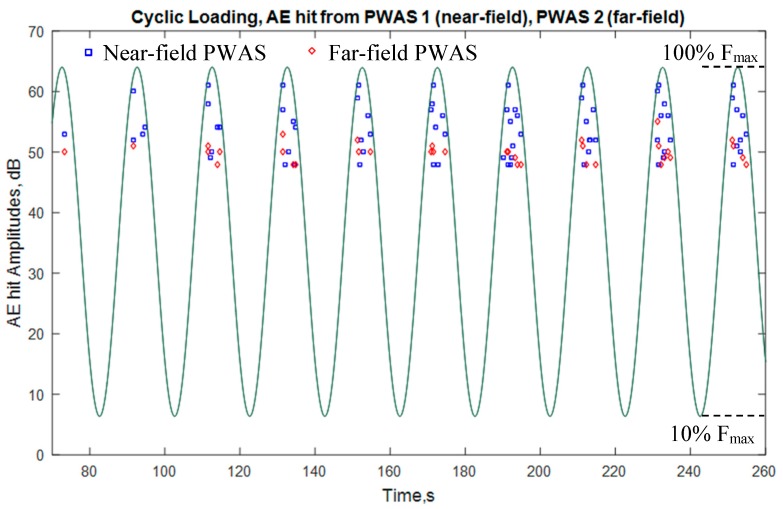
The AE-hits captured by the near-field and far-field PWAS transducers. The AE hits are plotted with the cyclic fatigue loading. The AE hits from the crack happened when the fatigue loading reached near the peak load (A portion of the total cycles was shown here for clear illustration of the AE hits per loading cycle).

The fatigue crack released multiple AE hits in each cycle as the crack grew. The AE hits captured by the PWAS transducers are plotted with the cyclic loadings on Figure 11. This shows that AE hits happened at almost every cycle of the fatigue loading. Most of the AE hits happened near the peak load. Because, when the load reaches to the peak load, the stress level at the tip of the crack reaches a critical level causing the failure of material-bonding and may cause the crack growth. A portion of the total cycles was shown in Figure 11 for clear illustration of the AE hits per loading cycle. It was observed that as the fatigue cycle continued more AE hits per cycle were captured by the PWAS transducers (not shown here). 

Figure 11 also shows that near-field PWAS captured more AE-hits than the far-field PWAS, as expected. The both sensors may capture the propagating AE signals. The near-field PWAS may capture the evanescent AE signals that would diminish as they travel further distance and could not meet the threshold of AE hit detection. The propagating AE signals showed geometric spreading and structural attenuation as they traveled far away from the crack. 

**Figure 12:**
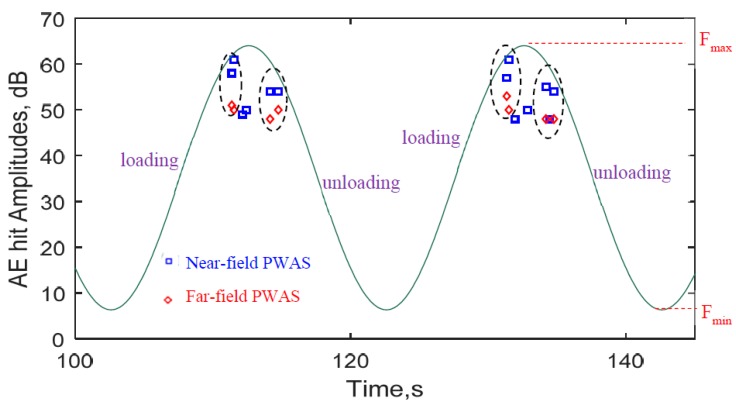
Zoomed-in view of the AE hits with the cyclic fatigue loading. The AE hits from the crack happened near the peak load level during both loading and unloading period of the cycle (PWAS 1 and PWAS 2 AE hits were distinguished by blue-square and red-rhombus legend, respectively).

Figure 12 shows the zoomed-in view of two fatigue cycles (100 s to 145 s) of Figure 11. This clearly shows the one-to-one correspondence between the AE hits captured by the two PWAS transducers. In the first cycle of Figure 12, the two AE-hits happened during loading period and both of them are captured by the two sensors. The similar observation can be made for the unloading period. However, there were two weaker AE hits that were captured by only near-field PWAS transducer. The similar observation holds true for the second cycle of Figure 12. Note that each AE hit corresponds to an AE signal.

### 5.2. Fatigue Crack Generated AE Signals Captured by the Two PWAS Transducers

**Figure 13:**
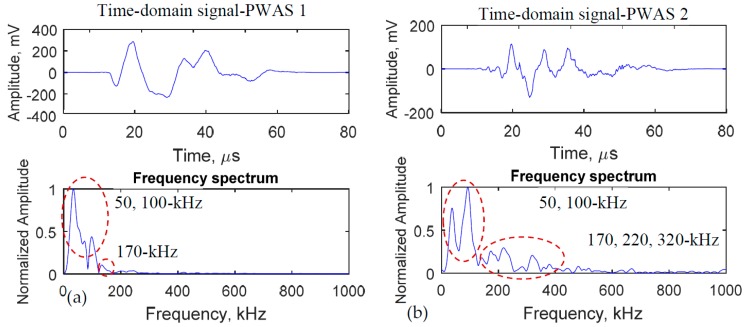
Experimentally measured AE signals by the two PWAS transducers. The typical AE signals coming from the same AE event captured by the (**a**) near-field PWAS; (**b**) far-field PWAS. Some high-frequency contents gained amplitude as the acoustic wave had traveled away from the crack.

The AE signals from the fatigue crack were captured by the two PWAS transducers. The time domain signals and their frequency spectra are illustrated in Figure 13. The same AE event captured by the two sensors is shown here. The same AE event was confirmed by the one-to-one correspondence of the AE-hits as depicted earlier in Figure 12. The amplitude of the near-field PWAS signal was higher than that of the captured the far-field PWAS signal, as expected. This is because of the geometric spreading of the acoustic wave. The frequency spectra of the two signals (Figure 13) show some considerable differences. 

The highest peak of PWAS 1 signal is located at 50-kHz frequency. There are also other peaks at 100-kHz and 170-kHz frequencies. On the PWAS 2 frequency spectrum, the highest peak is located at 100-kHz. There are also some other peaks at 50, 170, 220, 320-kHz frequencies.

### 5.3. The Effect of AE Sensor Location on the AE Signals

The AE sensor position has a significant effect on the captured AE signals. The geometric spreading of the acoustic wave signal has been observed. Overall, as the distance increases from the source, the AE signals become weaker. Sometimes, the AE signal may not be detectable if the sensor is too far from the source and the hit-amplitude falls under the threshold. Since the near-field PWAS was placed very close to the fatigue crack source, the local dynamics of the crack surface may affect the near-field AE signals. In the simulation, the crack resonance (local dynamics of the crack surface) phenomenon resulted in a low-frequency peak (50-kHz) in the AE signal of the near-field PWAS. In the experimental AE signal, the near-field PWAS may capture this local crack resonance phenomenon that resulted in a similar low-frequency peak (50-kHz). Because of the dominant low-frequency peak, the higher frequency peaks may look smaller in the plot. Thus the multiphysics simulation helped us to understand the experimental AE signals. 

## 6. Comparison between the Multiphysics Simulation and Experimental Results

The multiphysics simulation and the experimental study suggested that the PWAS transducer were capable of the sensing the AE signals. The experimental results show the agreement with the simulation results in the following aspects

### 6.1. The Lower Amplitude Signals as the AE Signals Travel Far Away from the Crack

The multiphysics simulation showed that the amplitude of the far-field PWAS AE signal was lower than the near-field AE signal. The same observation was found true for the experimentally measured AE signals. This is intuitive and expected for any guided wave generated from a point source or a thickness-wise distributed line-source in a plate (this is also true for bulk waves). Thus we may conclude that the AE signals travel as guided waves in the structure. This finding was well-supported by the other researchers [8]. Furthermore, we may construe that the experimentally measured AE signals were generated from the tip of the fatigue crack.

### 6.2. The Effect of the Local Dynamics of the Crack on the AE Signals

**Figure 14:**
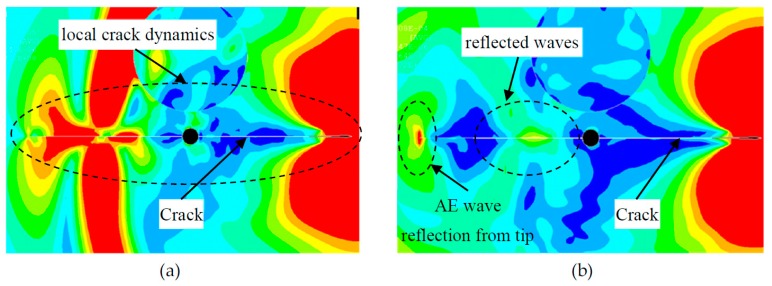
FEM simulation results of the (**a**) local dynamics of the crack; (**b**) reflection of AE waves from the crack tip.

The first two peaks of the AE signals were observed near 50-kHz and 100-kHz. Both multiphysics simulation and the experiment had supported these results. Note that the 50-kHz peak is dominant in the near-field sensor. Since the local crack dynamics was observed from the simulation (Figure 14), the similar phenomenon may happen during the experiment. This local dynamics may result in the 50-kHz peak in the experimental AE signals.

Also, this 50-kHz peak was becoming weaker as the AE signal travel far away from the crack but did not disappear as it reached to the far-field sensor. The effect of local dynamics (especially the secondary source from the other crack tip as observed from the simulation, Figure 14b) may propagate to the far-field sensor. However, it became weaker as observed from both experiment and simulation. On the other hand, the higher frequencies such as 170, 220, 320-kHz may be related to the plate guided waves. These frequencies may be related to the primary effect of the fatigue AE event at the crack tip.

### 6.3. Discussion of the Differences in Simulation and Experimental Results

In addition to the similarities between the simulation and experimental AE signal we have also observed some differences. The simulated near-field PWAS signal showed some frequency peaks at 200, 320-kHz. These frequency peaks were not so dominant in the experimental near-field PWAS signal. Also, on the far-field AE signal, the 450-kHz peak was observed in the simulation but not in the experimental signal. The several reasons could be responsible. For example, the exact features of the real fatigue crack were not the same as the simulation. These features include the zigzag nature of the actual fatigue crack propagation, through-thickness waviness, the slope of the fracture path that dictates the direction of the dipole force, and the actual amount of crack propagation in each AE event. Also, one component of moment tensor may not be enough to simulate the fatigue AE event as assumed in the simulation which would be considered for future investigation.

Despite the differences discussed above, the multiphysics simulation results were in very good agreement with the experimental results. The main objective that was to assess the capability of PWAS transducer to capture the low-amplitude fatigue crack related AE-signals was well-supported by both multiphysics simulation and the experimental measurement.

## 7. Conclusions

Piezoelectric wafer active sensors (PWAS) successfully captured the fatigue crack related acoustic emission signals. Both multiphysics simulations and experiments supported this result. The advancement of the fatigue crack generates the AE signals at every loading cycle. This indicates that the AE events happen at every cycle as the fatigue crack grows. The simulation results suggested that the dynamics of near-field PWAS had a minimal effect on the far-field PWAS with the prescribed distance mentioned in the paper. The effect of the AE event at the crack tip travels as guided acoustic waves. The AE signals show the geometric spreading of the amplitudes which is similar to the Lamb waves originating from a point source. The distance of the PWAS transducer has a significant effect on the sensing AE signals. The near-field PWAS captures the evanescent AE signals which resulted in higher number of AE hits in the near-field PWAS than that in the far-field PWAS. The local dynamics of the AE signals and the fatigue crack surface has been identified that could potentially be used for the fatigue crack length estimation. This has been supported by both FEM simulation and experimental measurement.

## 8. Future Work

The non-linear crack analysis would involve self-contact algorithms for crack modeling and could be investigated. The simulated AE signals and the actual experimental fatigue AE signals cannot be exactly matched or matched within some tolerance unless the exact features of the fatigue crack could be modeled. The exact features include the zigzag nature of the actual fatigue crack propagation, through-thickness waviness, the slope of the fracture path that dictates the direction of the dipole force, and the actual amount of crack propagation in each AE event. The AE-fatigue experiment would be performed using the PWAS transducers and the commercial AE sensors. The AE signals from the two types of sensors would be compared to each other. The AE signals corresponding to the larger fatigue crack could be recorded to see if there is any change in the AE signals. The AE hits could be further analyzed to find any possible groups of AE signals. The multiphysics simulation could be performed for other components of the moment tensors. The simulated AE signals could be compared for different moment tensor components.
